# Sedative-Hypnotic Effects of *Glycine max Merr.* Extract and Its Active Ingredient Genistein on Electric-Shock-Induced Sleep Disturbances in Rats

**DOI:** 10.3390/ijms24087043

**Published:** 2023-04-11

**Authors:** Minsook Ye, SunYoung Lee, Hyo Jeong Yu, Kyu-Ri Kim, Hyun-Jung Park, In-Cheol Kang, Soon Ah Kang, Young-Shin Chung, Insop Shim

**Affiliations:** 1 Department of Physiology, College of Medicine, Kyung Hee University, Seoul 02447, Republic of Korea; 2 Department of Food Science and Biotechnology, Kyonggi University, 154-42, Gwanggyosan-ro, Youngtong-gu, Suwon 17104, Republic of Korea; 3 BioChip Research Center, Department of Biological Science, College of Biological Science, Hoseo University, Asan 31499, Republic of Korea; 4 Innopharma screen Inc., Incheon 21988, Republic of Korea; 5 Department of Conversing Technology, Graduate School of Venture, Hoseo University, Seoul 06724, Republic of Korea; 6 Department of Biotechnology and The Research Institute for Basic Sciences, Hoseo University, Asan 31499, Republic of Korea

**Keywords:** *Glycine max Merr.*, electroencephalography (EEG), sleep, electric foot shock stress-induced insomnia, sedative-hypnotic activity

## Abstract

*Glycine max Merr*. (GM) is a functional food that provides many beneficial phytochemicals. However, scientific evidence of its antidepressive and sedative activities is scarce. The present study was designed to investigate the antidepressive and calmative effects of GM and its biologically active compound, genistein (GE), using electroencephalography (EEG) analysis in an electric foot shock (EFS)-stressed rat. The underlying neural mechanisms of their beneficial effects were determined by assessing corticotropin-releasing factor (CRF), serotonin (5-HT), and c-Fos immunoreactivity in the brain using immunohistochemical methods. In addition, the 5-HT2C receptor binding assay was performed because it is considered a major target of antidepressants and sleep aids. In the binding assay, GM displayed binding affinity to the 5-HT2C receptor (IC50 value of 14.25 ± 11.02 µg/mL). GE exhibited concentration-dependent binding affinity, resulting in the binding of GE to the 5-HT_2C_ receptor (IC50, 77.28 ± 26.57 mg/mL). Administration of GM (400 mg/kg) increased non-rapid eye movement (NREM) sleep time. Administration of GE (30 mg/kg) decreased wake time and increased rapid eye movement (REM) and NREM sleep in EPS-stressed rats. In addition, treatment with GM and GE significantly decreased c-Fos and CRF expression in the paraventricular nucleus (PVN) and increased 5-HT levels in the dorsal raphe in the brain. Overall, these results suggest that GM and GE have antidepressant-like effects and are effective in sleep maintenance. These results will benefit researchers in developing alternatives to decrease depression and prevent sleep disorders.

## 1. Introduction

Sleep is essential for sustaining human health and well-being, and disturbances in sleep can negatively affect both physical and psychological health. Disturbance of sleep leads to problems with homeostatic control and elevates weakness against inflammation-related and chronic diseases [1]. Because the stress systems take an initiating role in adjusting to a consistently changing and challenging environment, it is crucial to determine whether these systems are influenced by lack of sleep. The human body prompts protective processes in an accommodative effort to sustain homeostasis. Insomnia may occur if these protections result in failure. The stress response is a preserved survival mechanism, in which the stimulation of the hypothalamic–pituitary–adrenal (HPA) axis provides a central function. The activation of the HPA axis is initiated by the secretion of corticotropin-releasing factor (CRF) from neurons in the hypothalamic paraventricular nucleus (PVN), giving rise to the secretion of pituitary adrenocorticotropic hormone (ACTH). ACTH moves through the body and gives rise to the secretion of glucocorticoids by the adrenal glands. The activation of the HPA axis is exquisitely regulated, and CRF release is coordinated by other endocrine and autonomic circuits to achieve a unified stress response [2]. Dysfunction of the HPA axis has been involved in both endocrine and anxiety-related disorders [3].

During initial sleep, HPA axis activity is repressed without interruption. In the latter half of sleep, HPA secretory activity boosts, almost reaching the upper limit for the circadian rhythm instantly upon waking up, and the important activity of the HPA axis and sympathetic nervous system has an effect on the amount of rapid eye movement (REM) sleep [4]. The fact that the starting and finishing of sleep involves HPA axis activity and the close temporal relationship between the axis and sleep offers a key to understanding the effects of stress on sleep.

Stress is well known to be a common cause of short-term insomnia and induce sleep problems which are characteristics in the pathophysiology of stress-related disorders, such as depression, anxiety, or post-traumatic stress disorder (PTSD). Electric foot-shock (EFS) stress is utilized as a typical animal model which mimics the symptomology of stress-related disorders. It has been shown that exposure to electric shock reliably reduces both general and REM sleep, and foot shock is known as a more valid model of sleep changes in PTSD than immobilization stress [5,6]. Recent research demonstrates that foot shocks produce behavioral and neurochemical changes that reflect depression, anxiety, and PTSD in humans. For example, electric shock activates CRF and 5-hydroxytryptamine (5-HT) neurotransmission, as well as other systems inhibiting general and REM sleep [7].

Considerable evidence indicates the presence of brain 5-HT dysfunction in major depression [8] and sleep disorders. In addition, most antidepressants are thought to increase serotonergic neurotransmission. However, it is unclear whether the effect of sleep deprivation is due to enhanced serotonin.

The molecular targets for calmative effect primarily focus on modulating the serotonergic system, which is implicated in various mental functions related to relaxation, stress, and sleep regulation [9,10,11]. The 5-HT2A, 2B, and 2C receptors, mostly known to be coupled with G-protein, have been reported to play a pivotal role in various central nervous system functions [12,13]. In particular, the 5-HT2C receptor has been reported as a potential target for sedative or anxiolytic drugs [8].

*Glycine max Merr.* (GM) is a singular food source because of its rich nutrient content, including carbohydrates, proteins, isoflavones, minerals, and dietary fiber. GM intake has been linked to the prevention of diabetes mellitus, osteoporosis, skin disorders, and cancer. The health benefits of GM are attributed to the presence of bioactive compounds, which can be categorized into nonprotein compounds, such as isoflavones and saponins, and protein compounds. These compounds play a key role in several health effects owing to their ability to bind to estrogen receptors. Likewise, isoflavones from GM, such as genistein (GE), daidzein, and glycitein, have also shown immunomodulatory activity. GE, the most prominent component of GM isoflavones, possesses proverbial preventive activities against obesity, cardiovascular diseases, cancer, and liver injury. In addition, it may alleviate symptoms of menopause. However, no studies have examined the effect of GM and GE on sleep, and no conclusive mechanism for the clinical cure or sleep enhancement and associated disorders has been explicated.

The present study assessed the potential of GM extract and GE as a new treatment to reduce somnipathy in the EFS-stressed animal model in rats. The study aimed to demonstrate the effects of GM extract and GE on sleep and provide a basis for further research on the underlying mechanisms connected with a potential GM extract cure.

## 2. Results

### 2.1. Binding Affinity GM to 5-HT_2C_ Receptor

**Figure 1:**
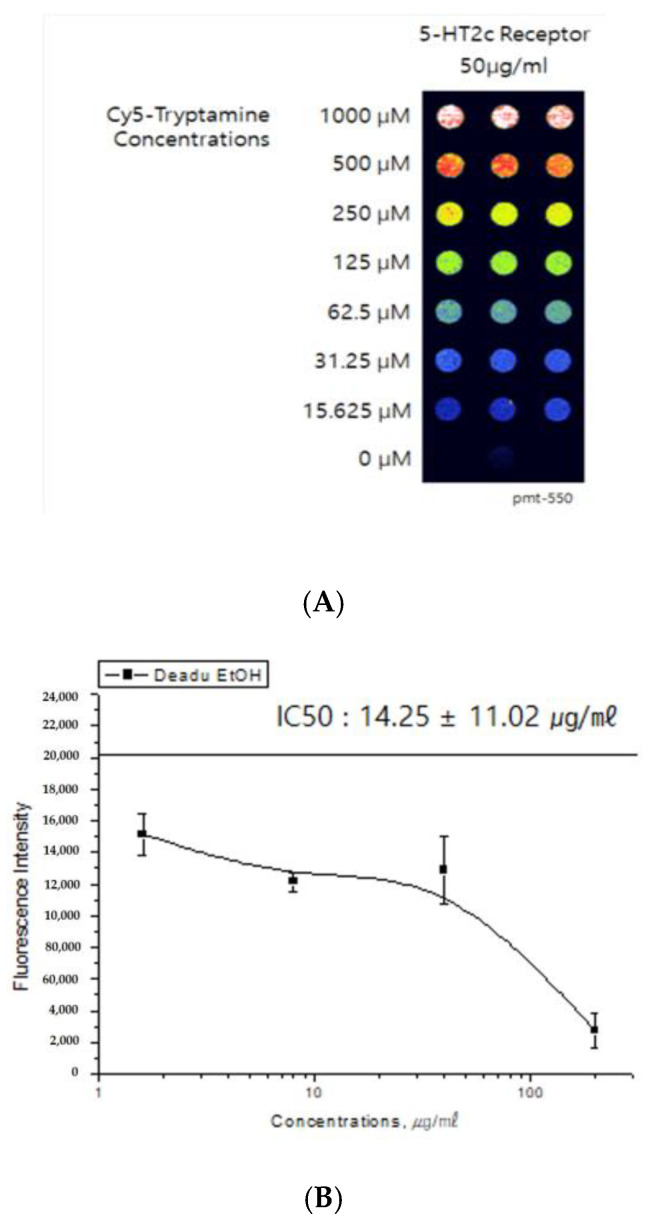

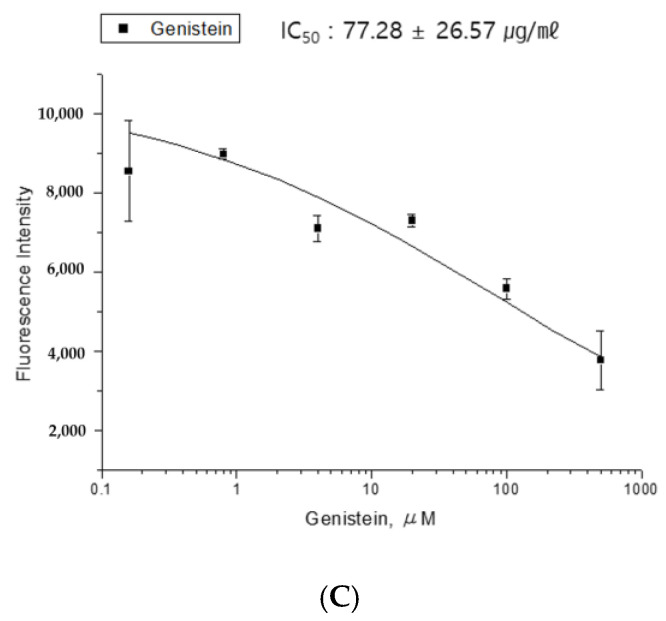
Dose–response curve and half-maximal inhibitory concentration (IC50) of GM (**B**) and GE (**C**) in the 5-HT2C receptor binding assay. For screening effective antagonists to the 5-HT receptor, GM and GE were reacted to a Protein Chip bound to 5-HT2c receptors, and then Cy5-tryptamine was added (**A**). Red color represents fluorescence intensity to Cy5-labeled tryptamine alone bound to 5HT2c receptors, and yellow color represents competitively drug-induced inhibitory binding activity.

The binding activity of GM to the 5-HT2C receptor was displayed as 5-HT2C agonist binding. We found that GM displayed effective binding activity (Figure 1). The inhibitory concentration (IC50) value of GM and GE were 14.25 ± 11.02 µg/mL (Figure 1B) and 77.28 ± 26.57 µg/mL (Figure 1C), respectively. Each concentration value of GE was 8535 ± 735.6 µg/mL, 8966.5 ± 73.08 µg/mL, 7097.7±187.4 µg/mL, 7287 ± 83.28 µg/mL, 5568.5 ± 150.64 µg/mL, and 3753.5 ± 424.99 µg/mL.

### 2.2. Effect of GM and GE on EEG Sleep Architecture and Profile

**Figure 2:**
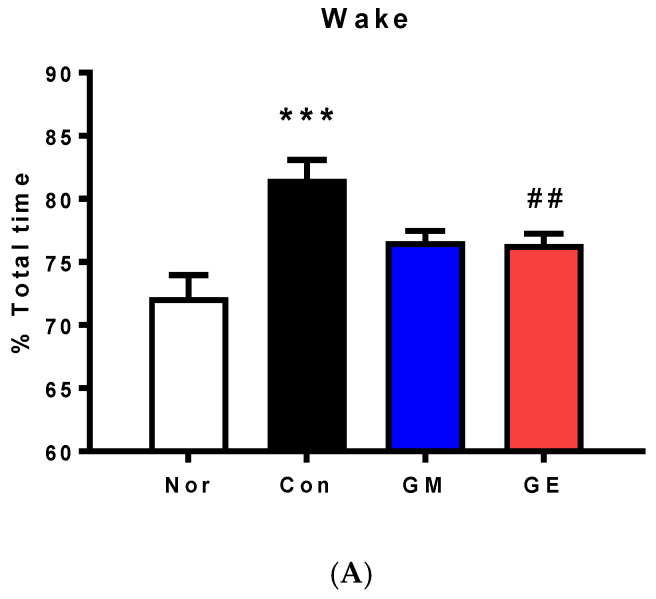

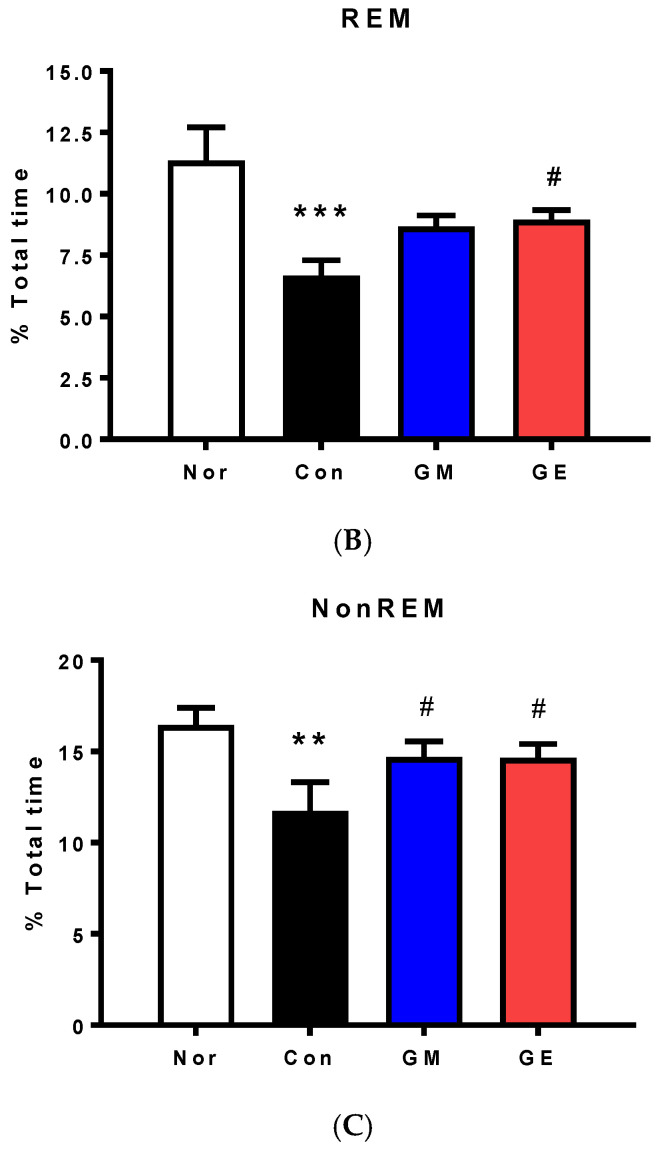
The effect of GM and GE on sleep architecture in the EFS-stressed rat. Changes in the percentage of wake (**A**), REM sleep (**B**), and NREM sleep (**C**) after GM and GE administration. The data represent the mean ± SEM of percent time spent in the sleep–wake state. ** *p* < 0.01, *** *p* < 0.001 vs. No (no stress); # *p* < 0.05, ## *p* < 0.01, vs. control (saline with EFS); one-way ANOVA followed by Tukey’s test. Normal (n = 6), control (n = 6), GM (n = 6), GE (n = 6).

The control group had a significantly increased wake time (81.57 ± 1.49) and decreased REM (6.67 ± 0.63) and non-rapid eye movement (NREM) (11.75 ± 1.57) compared to the normal group (Wake: 72.2 ± 1.76, REM: 11.36±1.35, NREM: 16.44 ± 0.94). We observed that EFS had negative effects on wake time (F (3,20) = 8.732, *p* < 0.001, Figure 2A), REM (F (3,20) = 5.741, *p* < 0.01, Figure 2B), and NREM (F (3,20) = 3.234, *p* < 0.05, Figure 2C) sleep times, causing disturbances of sleep. The GE group showed significantly increased REM and NREM sleep time compared to the control group (*p* < 0.05, Figure 2C). Additionally, the GM group showed significantly increased REM sleep compared to the control group (*p* < 0.05, Figure 2B). The GM groups did not produce any noticeable change in REM sleep; however, they did show a tendency to increase REM sleep time (Figure 2B). These results suggest that GE enhances wake time, NREM, and REM sleep and reduces EFS-induced loss of sleep, whilst GM only increases NREM sleep.

### 2.3. Effect of GM and GE on the CRF and c-Fos Immunoreactivity in the PVN

**Figure 3:**
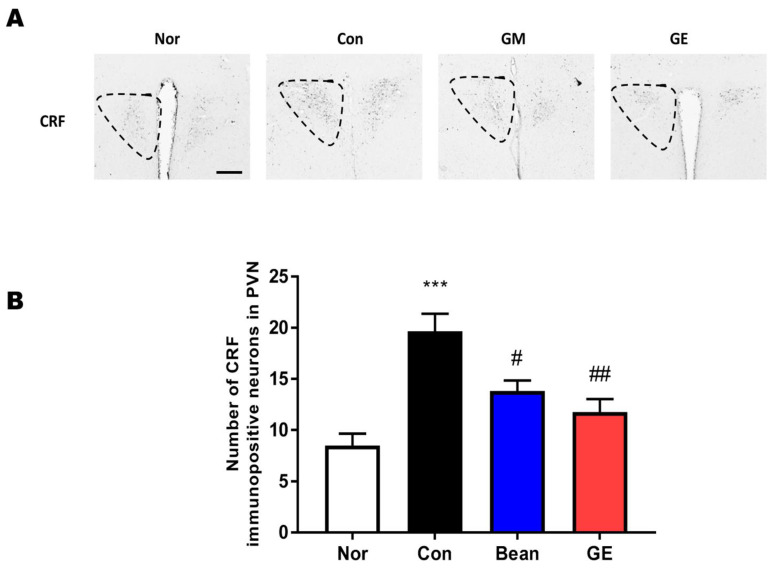
Effect of GM and GE on CRF neurons in the PVN. (**A**) Photomicrographs of CRF-positive cells in the PVN. (**B**) Number of CRF-positive cells in the PVN. *** *p* <0.001 vs. No (no stress); # *p* < 0.05, ## *p* < 0.01, vs. control (saline with EFS); one-way ANOVA followed by Tukey’s test. Normal (n = 12), control (n = 12), GM (n = 12), GE (n = 12). The scale bar represents 50 µm.

**Figure 4:**
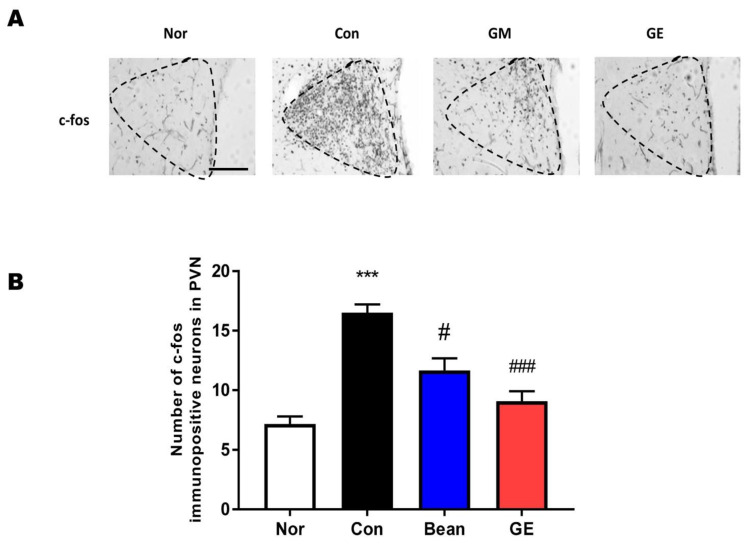
Effect of GM and GE on c-Fos activated neurons in the PVN. (**A**) Photomicrographs of c-Fos activated neurons in the PVN. (**B**) Number of c-Fos activated neurons in the PVN. *** *p* < 0.001 vs. No (no stress); # *p* < 0.05, ### *p* < 0.001, vs. control (saline with EFS); one-way ANOVA followed by Tukey’ test. Normal (n = 12), control (n = 12), GM (n = 12), GE (n = 12). The scale bar represents 100 µm.

The expressions of CRF and c-Fos in the brain were assessed in all four groups (Figure 3 and Figure 4). Previous studies have shown that c-Fos and CRF levels are increased after EFS. We evaluated CRF expression in the PVN (F (3,44) = 2.64, *p* < 0.001, Figure 3) and found that the amount of CRF immunoreactivity was lower in the GM and GE groups than that in the control group (*p* < 0.05, *p* < 0.01, respectively, Figure 3. The expression of PVN c-Fos was measured in the GM and GE groups (F (3,44) = 25.37, *p* < 0.001, Figure 4). Compared with the control group, the expression of c-Fos in the normal group was significantly lower. The GM and GE group showed dramatically decreased levels of c-Fos in the PVN region of the brain compared to the control group (*p* < 0.05, *p* < 0.01, respectively, Figure 4). These results suggest that GM and GE may enhance sleep patterns by decreasing neuronal activity in sleep-related regions of the brain, as detected by downregulated c-Fos immunoreactivity.

### 2.4. Effect of GM and GE on 5-HT Level in the Dorsal Raphe Nucleus

**Figure 5:**
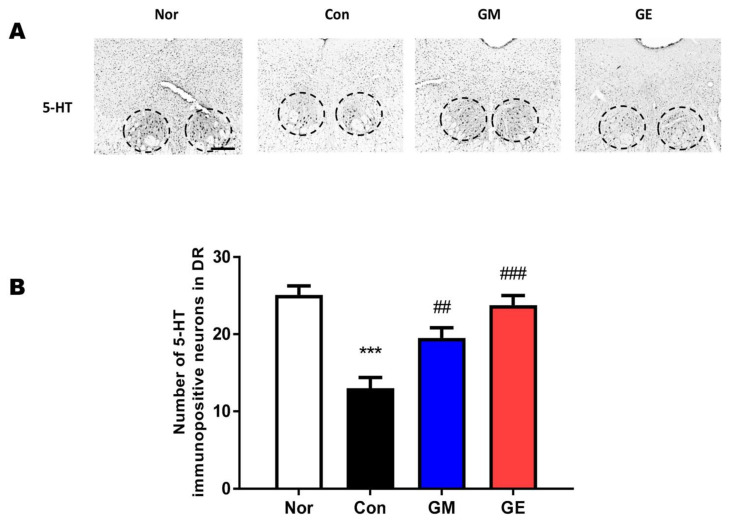
Effect of GM and GE on 5-HT-positive cells in the DRN. (**A**) Photomicrographs of 5-HT-positive cells in the DRN. (**B**) Number of 5-HT-positive cells in the DRN. *** *p* < 0.001 vs. No (no stress); ## *p* < 0.01, ### *p* < 0.001, vs. control (saline with EFS); one-way ANOVA followed by Tukey’s test. Normal (n = 12), control (n = 12), GM (n = 12), GE (n = 12). ). The scale bar represents 100 µm.

The level of 5-HT in the brain was assessed in the GM and GE groups (Figure 5). The GM group showed considerably upregulated 5-HT levels in the dorsal raphe nucleus (DRN) region of the brain (F (3,44) = 17.74, *p* < 0.001, Figure 5). The GE group showed dramatically upregulated levels of 5-HT in the DRN region of the brain (*p* < 0.001, Figure 5).

## 3. Discussion

Several regions of the brain are involved in modulating sleep. Monoaminergic neurons, in particular, are involved in the conversion of the sleep–wake cycle and the balance of sleep patterns in various regions of the brain [14]. The PVN, situated in the ventral diencephalon adjacent to the third ventricle, is involved in numerous neurobiological processes. CRF neurons are the most abundant in the hypothalamic PVN region and play a crucial role in the adaptation of the body to stress. They co-ordinate stress-related behaviors through direct projections to the limbic and autonomic brain systems. The DRN provides the majority of 5-HT throughout the central nervous system [15]. Serotonergic neurons in the DRN play an important role in sleep–wake regulation [16]. Most serotonergic neurons in the DRN fire regularly at a slow rate during wakefulness, fire considerably less during NREMS, and even cease firing during REMS. These sleep mechanisms were the foundation of the present study. We aimed to first check the classic sleep-related signal pathway, such as the serotonergic and PVN CRF, c-Fos expression, and the sleep enhancement and preservation effects of GM and GE.

A receptor-binding assay is an important tool in the search for drug candidates. In particular, the 5-HT2C receptor binding assay has been extensively used to screen for calmative and anxiolytic activities. GM displayed a moderate dose-dependent binding affinity to the 5-HT2C receptor (IC50 value of 46.62 µg/mL). This result indicates that GM contained natural ligand-binding affinity to the 5-HT2C receptor.

The present study evaluated the effects of GM and its bioactive component GE on sleep enhancement, induction, and preservation of stress-induced sleep interferences. We found that GM and GE reduced the wake ratio by sustaining a balance between sleep patterns and influencing the expression of PVN c-Fos and CRF in EFS-induced sleep disturbance. Additionally, GM and GE significantly increased REM time. GM and GE decreased CORT serum concentrations and maintained better sleep by increasing 5-HT immunoreactivity in the dorsal raphe in an ESP-induced sleep model.

Foot-shock stress is widely utilized as a model to mimic the symptomology of stress-related disorders in animals. Previous studies have shown that exposure to electric shock reliably reduces general and REM sleep. We confirmed that EFS significantly increased total awake time, and reduced REM and NREM time, as seen in Figure 2A–C, which is consistent with the earlier research.

We identified GE as a bioactive constituent of GM and confirmed its effects on sleep enhancement and maintenance. In this study, GE showed similar effects to GM in the EFS-induced sleep disorder animal model. These results suggest that GM extract and GE may be effective in sleep maintenance and enhancement.

Stress is a common cause of short-term insomnia, and the close relationship between sleep and stress has recently been identified. Sleep is commonly evaluated in many studies by exposing animals to foot-shock parameters planned to induce stress. GM and GE treatment after EFS exposure decreases serum corticosterone levels, the amount of c-Fos- and CRF-immunoreactivity in the PVN, and the level of 5-HT in the DRN. Our results indicate that GM and GE can mitigate the stress that causes disturbances of sleep.

Changing electroencephalogram (EEG) frequencies help estimate sleep–wake states. During wake time, the EEG recordings go up and down by high-frequency and low-amplitude EEG. The power of EEG is considerably decreased in the low-frequency δ-wave and increased in the θ-wave range during REM sleep. NREM sleep is scored based on the presence of spindles interspersed with slow waves. In the sleep pattern of rats exposed to EFS, wake times increase and REM sleep time decreases until 12 h after sleep. Similarly, in this study, we induced EFS-related sleep disturbances by increasing wake times and decreasing REM sleep times. Our findings suggest that GM and GE stabilize sleep patterns by restoring wakefulness and REM sleep ratios.

Previous studies have suggested that treatment with herbal medicine, which exhibits hypnotic effects, altered total sleep time and NREM rather than REM sleep [17]. With these results in mind, the present study showed that GM and GE reduced wake times and promoted significant NREM sleep. Although the central serotonergic system has been implicated in promoting wakefulness [18], its exact role in the sleep–wake system remains controversial. In previous studies, extracellular 5-HT release during sleep deprivation was decreased [19]. 5-HT was reported to enhance sleep [20]. In the present study, 5-HT levels in the DRN were increased in both the GM and GE groups.

We found enough evidence that GM and GE enhance stress and quality of sleep by studying some parameters, such as EEG, PVN c-Fos, CRF immunoreactivity, and DRN 5-HT, in an animal model of EFS-induced sleep. GM affected the 5-HT expression and the number of c-Fos- and CRF-immunoreactive neurons and restored NREM sleep patterns and CORT levels, which as a collective acted to reduce stress and is the major cause of somnipathy. GE had a particularly significant effect on wake times, REM, NREM, 5-HT expression, and the number of c-Fos- and CRF-positive cells in the PVN, thus supporting the action of GM on sleep improvement.

Based on the sleep enhancement effects of GM and GE in animals, we intend to introduce GM and GE as treatments or supplements for sleep disturbance. In this study, to verify the effects of GM on sleep in an animal model, representative parameters, such as EEG, c-Fos, 5-HT, and CRF, were analyzed. In the model of EFS-induced sleep disturbance, the effectiveness of GM and GE was confirmed for Wake/REM sleep cycles and c-Fos and CRF levels were decreased. In addition, 5-HT expression levels were increased. The present study demonstrated that GE binds to 5-HT2C receptors and synergistically stimulates serotonergic neurons in the DRN. The serotonergic systems may be heavily involved in modulating the sedative or anxiolytic-like effects of GE. In addition to these studies, we also showed that GE reduced wake time and promoted significant REM and NREM sleep through the serotonergic system. These results suggest that GE produces its hypnotic and anxiolytic-like effects through the interaction of the 5-HT2c receptors, which are known to play an important role in sleep functions.

Overall, these results suggest that GM and GE have antidepressant-like effects and are effective in sleep maintenance. These results will benefit researchers in developing alternatives to prevent depression and sleep disorders.

## 4. Materials and Methods

### 4.1. Preparation of GM and GE, and HPLC Analysis

Soybeans (GM, 3000 g) were powdered and made circumfluent with 30% ethanol (3 times the weight of the beans) for 3 h at 90 °C. The ethanol supernatants were percolated and lyophilized to yield the final extract (362 g, 12.1%). GE was supplied by Sigma–Aldrich. (Sigma–Aldrich, Saint Louis, MO, USA).

High-performance liquid chromatography (HPLC) was performed using Agilent 1100 series G1379A Degasser, G1312A Bin Pump, G1313A ALS, G1316 column, and G1313A VWD (Agilent Technologies, Santa Clara, CA, USA). As the HPLC column, a Luna (18, 150 × 4.6 mm, 5 μm) column was used, and the wavelength of the ultraviolet absorption detector was 254 nm. As the moving phase, an ammonium sulfate solution (1 → 100) and acetonitrile mixture solution (91.5:8.5, *v*/*v*) were used. Thereafter, 10 μL of the sample was injected.

**Figure 6:**
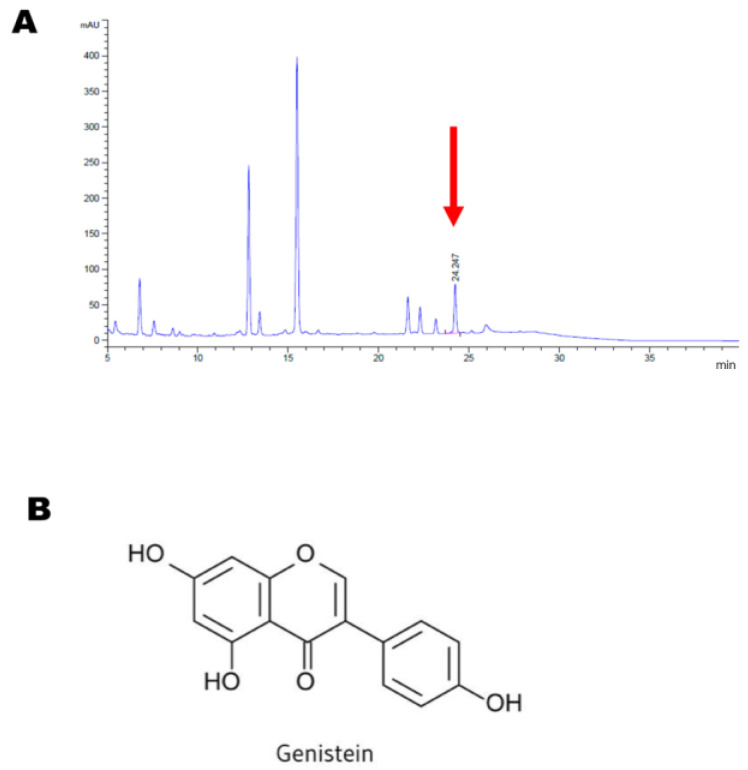
The HPLC analysis of the standard material to GM. Phytochemical analysis was performed by HPLC as described in the materials and methods section. (**A**) GE was utilized as an authentic standard (arrows), and the concentration of GE in GM was calculated. (**B**) Chemical structure of GE.

Using this method, the concentration of GE in GM was calculated to be 7.74 µg/g, as described in Figure 6.

### 4.2. 5-HT2c Receptor Binding Assay

HEK293 cell lysate (100 μg) was labeled with Cy5 (GE Healthcare Bio-Sciences Corp., Marlborough, MA, USA) and free dyes were abandoned by Spin column (Sigma Aldrich, Saint Louis, MO, USA). To make 5-HT2c receptor microarray, a glass slide covered with super epoxy group (Arrayit Corporation, Sunnyvale, CA, USA) was used. The antibody microarray using the glass chip was prepared by immobilizing 42 antibodies against cell cycle proteins. Cy5-labeled tryptamine and GM were applied to the Protein Chip and incubated for 1 h at 37 ◦C. The Protein Chip was then washed with PBST and DW and dried under a stream of N2 gas. GM was dissolved in ethanol and adulterated to the desired concentration using PBS. The GM concentrations ranged from 1000 µM to 15.625 µg/mL. Tryptamine alone was used as the negative control. GM was used to estimate its calmative effect using a 5-HT2c receptor binding assay. The chips were scanned using a GenePix 4100 A microarray scanner (Axon Instruments, Union City, CA, USA). The internally normalized proportion of all spots was evaluated.

### 4.3. Animals

Eight-week-old SD rats from Samtako Animal Co. (Osan-si, Gyeonggi-do, Korea) were used for this experiment. The system automatically maintained proper temperature (20–25 °C), humidity (45–65%), and lighting (12:12 h light/dark cycle). Food and water were supplied ad libitum. All animal experiments were approved by the Institutional Animal Care and Use Committee of Kyunghee University Health System (KHUASP (SE)-14-051).

The rats were split into four groups of six rats each, according to the EFS-induced sleep disorder model. The four groups were as follows: normal (“nor”: no stress), stress/control group (“control”: saline with EFS), “GM” (400 mg/kg of GM extract with EFS), and “GE” (30 mg/kg of GE with EFS).

### 4.4. Sleep-Related Animal Model and Drug Administration

After electroencephalogram (EEG) operation, the animals received EFS once a day for 5 days. EFS was administered in accordance with the following conditions at random: frequency = 10 times in 3 s; intensity = 3 mA; duration = 5 min.

### 4.5. EEG Surgery

Electrodes of EEG were transplanted for polygraphic recording as described in the stereotaxic atlas of Paxinos and Watson [21]. Anesthetic operation was performed using pentobarbital (40 mg/kg, i.p.), after which the rats were chronically transplanted with a head mount. The electrodes were anchored into the skull with screws and dental cement. All surgical procedures were conducted stereotaxically under aseptic conditions. After the operation, all rats were permitted to restore for 7 days and observed closely for any signs of pain, such as reduced appetite or decreased bodyweight.

### 4.6. Methods of the EEG Recording

Animals were habituated to the recording conditions prior to the test after the operation. GM and GE were separately dissolved in 0.9% saline solutions (GM concentration = 400 mg/kg, GE concentration = 30 mg/kg) and administered orally for 5 days, as well as administering EFS stress before EEG recording. Treatment with saline and bean GE was performed 10 min before the EEG recording. After treatment, animals were immediately connected to EEG recording cable (two EEG channels). The software indicates wakefulness as high-frequency low-amplitude EEG, and NREM was scored on the presence of spindles scattered with slow waves in the EEG. EEG power during REM was significantly decreased in lower-frequency δ-wave (0.75–4 Hz) and increased in the range of θ-wave activity (5.0–9.0 Hz, peak at 7.5 Hz).

Rats were habituated to the recording conditions prior to the test after operation. GM and GE were separately dissolved in 0.9% saline solutions (GM concentration = 400 mg/kg, GE concentration = 30 mg/kg) and administered orally for 5 days and EFS stress was administered before EEG recording. Treatment with saline and bean GE was performed 10 min before the EEG recording. After treatment, rats were immediately connected to EEG and EMG recording cables (two EEG channels and one EMG channel). The software discriminates wakefulness as high-frequency low-amplitude EEG, and NREM was scored on the basis of the presence of spindles interspersed with slow waves in the EEG. EEG power during REM was significantly reduced in lower frequency δ-wave (0.75–4 Hz) and increased in the range of θ-wave activity (5.0–9.0 Hz, peak at 7.5 Hz).

### 4.7. Immunohistochemistry

The brain tissue samples were fixed in 4% formaldehyde (Sigma Aldrich, Saint Louis, MO, USA) and 30 µm coronal sections were in 0.3% hydrogen peroxide (Sigma Aldrich, Saint Louis, MO, USA) for 10 min to eliminate endogenous peroxidase activity and washed with phosphate-buffered saline containing 0.2% tween-20 (PBST). The brain tissue samples were incubated with 3% BSA (Thermo Fisher Scientific, Inc., Waltham, MA, USA) and 0.2% triton x-100 at room temperature (RT) for 1 h to block nonspecific binding; then, brain tissue samples were rinsed with PBST and incubated at 4 °C overnight with primary antibodies against CRF (1:500), c-Fos (1:1000), and 5-HT (1:500). The following day, the tissues were washed with PBST and incubated with a secondary antibody (1:500; rabbit; Vector Laboratories, Burlingame, CA, USA) at RT for 2 h. The signal was visualized using an ABC kit (Vector Laboratories, Burlingame, CA, USA) and DAB substrate kit (Vector Laboratories, Burlingame, CA, USA). After washing with PBS for 10min, the sample tissues were imaged using a light microscope.

### 4.8. Statistical Analysis

All results were analyzed using IBM SPSS 23.0 (Armonk, NY, USA) and presented as mean ± standard error of the mean (SEM). Statistical comparisons were performed for the EEG and immunological studies using one-way analysis of variance (ANOVA), followed by Tukey tests. Differences were considered significant at a *p*-value ≤ 0.05.

## Data Availability

The data used to support the findings of this study are available from the corresponding author upon request.
